# The trend of change in the role of pre-hospital emergency medical services in Iran’s healthcare system: a situational analysis

**DOI:** 10.1186/s12873-023-00861-3

**Published:** 2023-08-30

**Authors:** Kheizaran Miri, Mohammadreza Sabbaghi, Seyyed Reza Mazlum, Mohammad Namazinia

**Affiliations:** 1 Department of Nursing, School of Nursing and Midwifery, Torbat Heydariyeh University of Medical Sciences, Torbat Heydariyeh, Iran; 2 Department of Medical Emergency, School of Nursing and Midwifery, Torbat Heydariyeh University of Medical Sciences, Torbat Heydariyeh, Iran; 3 Department of Medical - Surgical Nursing, School of Nursing and Midwifery, Mashhad University Medical of Medical Sciences, Mashhad, Iran

**Keywords:** Situational Analysis, Grounded Theory, Role, Pre-Hospital Emergency Medical Services, EMS, Iran

## Abstract

**Introduction:**

Following the significant changes in healthcare systems over the last century, the role of pre-hospital emergency medical services (EMS) has been drawn against numerous challenges. In view of this, the present study aims to reflect on the trend of change in the role of pre-hospital EMS to meet new situations and needs, thereby providing a clear picture of this process.

**Methods:**

Respecting the intricate concept of development and the major changes in Iran’s healthcare system, this study was fulfilled using situational analysis (SA), developed by Clarke (2018), in Iran within 2022–2023. For this purpose, the data were collected through semi-structured, in-depth individual interviews with four faculty members, two managers involved in EMS, and six highly skilled EMS personnel in various positions. Moreover, the relevant articles published from 1950 to 2023 were reviewed. The data analysis was then performed via SA in the form of open coding as well as simultaneous analysis through three types of maps, viz., situational, social worlds/arenas, and positional maps, along with constant comparative analysis.

**Results:**

Pre-hospital EMS in Iran has thus far had two turning points from 1970 to 2023, wherein academic components, need for equipment and resources, in conjunction with basic needs in the modern society, have been the main propellers. As well, the complexity of care for non-communicable diseases (NCDs), demand for managed care, and technological advancement has gradually resulted in further development in EMS. This trend describes the EMS situation in Iran from 1973 to 2023, as well as the EMS emergence stages, quantitative growth and infrastructure, and clinical education development in 2002–2023, indicating the lack of funding and inadequate scientific infrastructure in proportion to the population receiving such services.

**Conclusion:**

Considering the trend of change in the approaches adopted by healthcare systems across the world, and given the breakthroughs in nursing and medicine, along the education of professionals during the last thirty years, the descriptions of duties and performance in EMS have moved from primary care and patient transfer to specialized services and outpatient care. In addition, the cultural context specific to Iran, the challenges of women working in EMS centers, the disconnection of service providers, namely, the Iranian Red Crescent Society (IRCS) Relief and Rescue Organization (R&RO), Iran’s National Police Force (INPF), and Iran’s National Medical Emergency Organization (INMEO), as well as lack of resources and equipment, and the geographical distribution of human resources (HRs) based on population dispersion, are thus among the significant issues facing pre-hospital EMS in this country.

## Introduction

Pre-hospital emergency medical services (EMS) are known as the key element in healthcare systems around the world, since they play a vital role in medical teams [1, 2]. The major changes in such services can thus affect the safety and quality of care to clients. Considering the current advances in medical sciences, complicated care for non-communicable diseases (NCDs), and limited resources in the world, EMS provision requires the classification of care and educational programs and courses [3–6]. EMS education is accordingly the first level and the shortest pre-hospital alternative in terms of duration, curriculum content, and costs, which helps provide the primary services, including vital sign measurement, trauma triage, first aid, basic or advanced cardiopulmonary resuscitation (CPR), etc. [7–9].

Notably, EMS emerged in developed countries in the 1970s. With the trend of change in the role of EMS from 1973 to 2023 worldwide, it has accordingly moved from primary care and patient transfer to specialized services and outpatient care [10]. In 1973, numerous people were injured and killed when the roof of the main hall of the Mehrabad International Airport terminal building collapsed, but there was no system to aid and transport the injured. Following this disaster, Iran’s National Medical Emergency Organization was established as the fourth one in the world with help from Americans [11].

Having a population of about 88 million in southwestern Asia, Iran is a member of the World Health Organization (WHO) in the Eastern Mediterranean region [12–14]. Currently, Iran has 20000 pre-hospital EMS personnel involved at operation, communication, staffing, and education levels. The clinical setting for the provision of such services is also provided in 3000 centers (viz., 1700 road centers, 1300 urban centers, and 50 Air medical emergency centers) [15]. EMS have role in the all of the phases of disaster management including preparedness, response, and recovery. Within a given geographic region, the local EMS medical director and administrator are well positioned to strengthen the function of EMS through participation and collaboration in all phases of management with other responding agencies and sectors in a community or region in disaster [8, 16–18]. It is effective to do a systematic assessment of the healthcare sector especially prehospital system can be have important role in performing risk disaster management process [19]. Like other parts of the world, the role of EMS has been to meet the urgent needs of the society in this country, but Iran has encountered big changes in the quantity and quality of these roles that have been specific to its situation [20–22]. Many studies have so far shed light on the role of EMS and its development all over the world. Even if some components introduced for this purpose have been common in all countries, the diversity of roles implies the appropriateness of each one for specific needs with regard to its context and time. As well, the recognition and generalizability of these roles in all times, societies, and countries have not been possible, because they have been bound by numerous variables as well as sociocultural and political factors [23]. Therefore, the question raised in the present study is focused on the trend of change in the role of EMS, along with the factors and variables affecting this process, which are addressed here.

## Methods

### Design

**Fig. 1:**
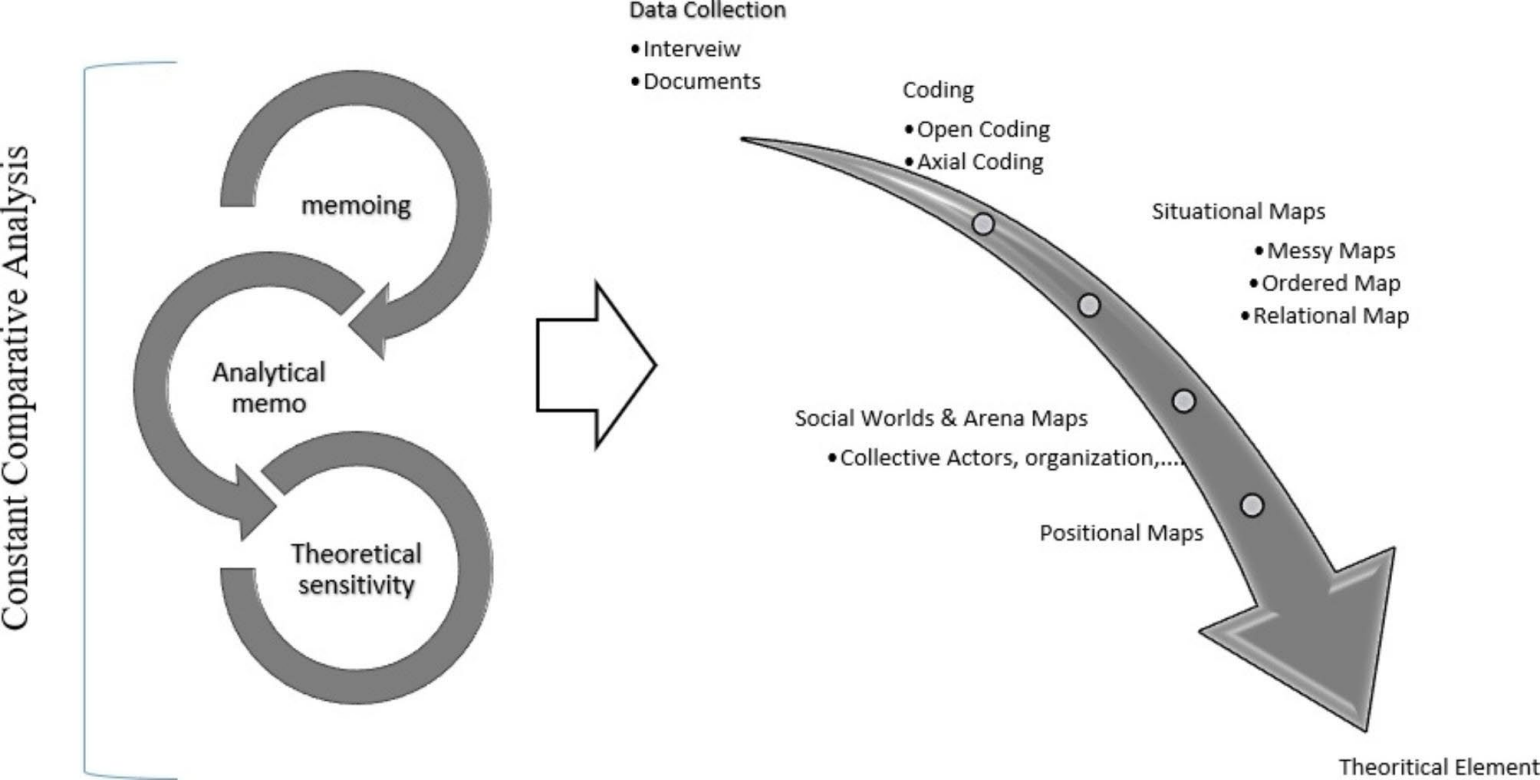
Process in Clarke’s situational analysis method

This study utilized grounded theory (GT) as a qualitative method with reference to situational analysis (SA), developed by Clarke (2018). Originating from GT, SA could thus make it possible to identify and describe the situational and positional complexities of social worlds and arenas through mapping [24]. As healthcare systems, with their dynamic and complex nature, help implement community health programs, but the complicated concept of role development in such systems heavily depends on various sociocultural and political variables and components, SA could be a good approach through analyzing three types of maps, namely, situational, social worlds/arenas, and positional ones, in this field (Fig. 1).

### Participants

This SA was conducted in Iran with the participation of pre-hospital EMS personnel in the related centers, the Medical Emergency and Accident Management Center (MEAMC) at the universities of medical sciences, the specialty boards (i.e., emergency medicine specialists, disaster and emergency specialists, and nurses), the Iranian EMS Association (IEMSA), and teachers at the schools of nursing and paramedical sciences, including the personal experiences and archives of Javad Malekzadeh, as one of the influential people in the last six decades of nursing in Iran, who has been working on policymaking, management, education, and research in this field from 1951 to 2019 (before and after the Islamic Revolution), and five key people of his era. Inclusion criteria included: willingness to participate in the study and having at least 10 years of work experience in various fields of pre-hospital emergency. Exclusion criteria: unwillingness to participate in the study or withdrawal from the study during the interview.

In total, 12 participants, varying in terms of gender, age, and academic degrees, were recruited (Table 1).

**Table 1:** participant demographic profile

Item	Response	N
Gender	Female	2
	Male	10
Age	Mean	55.3
	Median	57.0
	Mode	57.0
	Std. Deviation	14.7
	Range	59.0
	Minimum	26.0
	Maximum	85.0
Setting	Emergency organization	-
	Board Of Pre-hospital Emergency Care	-
	Association of Medical Emergencies	-
	Deputy of nursing	-
	Nursing Organization Of Islamic Republic of Iran	-
	Nursing and Midwifery School	-
	Pre Hospital	-
Human resourses(HR)	Faculty (Ph.D.).	2
	Faculty (MS)	2
	Policy maker(Ph.D.)	2
	Registered Nurse (RN)	2
	BSc Pre-hospital Emergency Care	2
	District Officer	1
	Area manager	1
Total		12

### Data collection

Using snowball sampling method, the EMS personnel were selected from winter 2021 to spring 2023, after obtaining their informed consent. Interview questions were designed based on research objectives and research literature review. Afterward, semi-structured, in-depth individual interviews started with the following questions (Table 2).

**Table 2:** In-depth and semistructured interviews questions.

Nnumber	question
1	What is the current state of EMS? How do you perceive the trend of change in the role of EMS in Iran? What developments have so far occurred?
2	What people and factors have played a pivotal role in these changes?
3	What are the concerns related to the role of EMS? What are the outcomes of the changes in the role of EMS?
5	How have managers acted during this process?

The interviews were conducted after participants read and understood. The locations of conducting interviews were chosen based on the ease and convenience of each participant. A relaxed setting, comprising 2 chairs and a small table between each participant and interviewer, was maintained. The interviews were recorded, which were kept strictly confidential. Additionally, personal space was ensured to listen to participants clearly without interfering in their space. The room where interviews were conducted quiet, had minimal distractions, sufficient lightning, and adequate temperature. Each interview took approximately one hour.

Upon answering each question, some follow-ups such as, *Can you explain* more, etc., were further addressed for exploratory purposes. Some interviews were also completed during two sessions in line with the participants’ tolerance, working conditions, and necessities. Each interview was initially documented with a digital audio recorder, and then transcribed into a Word file. From the fifth interview onward, repetitions were detected in the data, and they reached theoretical saturation from the sixth interview, but this process continued until the 12th interview for more assurance. Along with the interviews, the data were collected with reference to the documents from Dr. Madah Foundation (established in 2018 at the School of Nursing, affiliated to Tehran University of Medical Sciences, Tehran, Iran, where the historical documents of Iranian nursing from 1921 to 2021 are being archived), and the National Library and Archives of Iran (NLAI), including available statistics, government documents, international reports and historical information, the articles related to INMEO and the Iranian Nursing Organization (INO), and the studies about the role of EMS published from 1950 to 2023 in the databases of Web of Science, Scopus, and ScienceDirect, as well as the memos on the role of EMS.

### Data analysis

The analysis and initial coding of the data retrieved from each interview were performed before the next one. With regard to the qualitative data obtained from the interviews and the existing documents, the memos were thus analyzed after the data conversion. Data analysis was performed by the first author. In this case, like GT, simultaneous data analysis was done together with data collection, coding, and classification as a constant comparative process, and memoing. Although SA, developed by Clarke (2018), was similar to the Straussian approach for open and axial coding, it lacked selective coding and an axial variable to explain the theory. Along with the axial coding, the analysis was fulfilled via a triple approach of data mapping, using situational, social worlds/arenas, and positional maps. According to these data, theoretical elements, including situational maps (viz., human and environmental components and their connections), social worlds/arenas maps (namely, important non-human factors and components, and different arenas), and positional maps (i.e., responses of factors and reactions given or not given) were obtained.

### Trustworthiness

To establish the robustness of the study findings, the four stringent evaluative criteria (namely, credibility, confirmability, dependability, and transferability), proposed by Lincoln and Guba (1985) were implemented. With regard to the credibility of the findings, all codes extracted from each interview were shared and checked with the interviewees and then modified if necessary. Besides, over one year was devoted to data collection and analysis. To enhance the confirmability of the findings, the transcribed interviews together with the codes and categories extracted were reviewed, and then approved by the second and third authors as well as a faculty member outside the research team. For the dependability of the findings, the research stages and processes were recorded and reported as precisely as possible step by step. There were also attempts to observe maximum diversity in the participants in terms of service delivery location, EMS role, work experience, and job position, which helped augment the credibility and then the transferability of the findings. To check the accuracy of the documents, the historical data obtained from the primary sources were compared, and supplementary interviews were further conducted with informants. Considering the external criticism of the documents, library documents as the secondary sources and their details were compared with other sources (such as interviews and other documents) and analyzed. Regarding the internal criticism of such documents and the authenticity and validity of their content, given that the primary sources were still available, historical informants were also recruited.

## Results

In this study, the trend of change in the role of EMS in Iran over the last century was described by SA, using situational, social worlds/arenas, and positional maps. The description of the situation of the role of EMS in Iran from 1973 to 2023 included the following steps, viz., EMS emergence, quantitative growth and infrastructure, and clinical education development in Iran in 2002–2023, indicating the lack of funding and inadequate scientific infrastructure in proportion to the population receiving EMS.

**Fig. 2:**
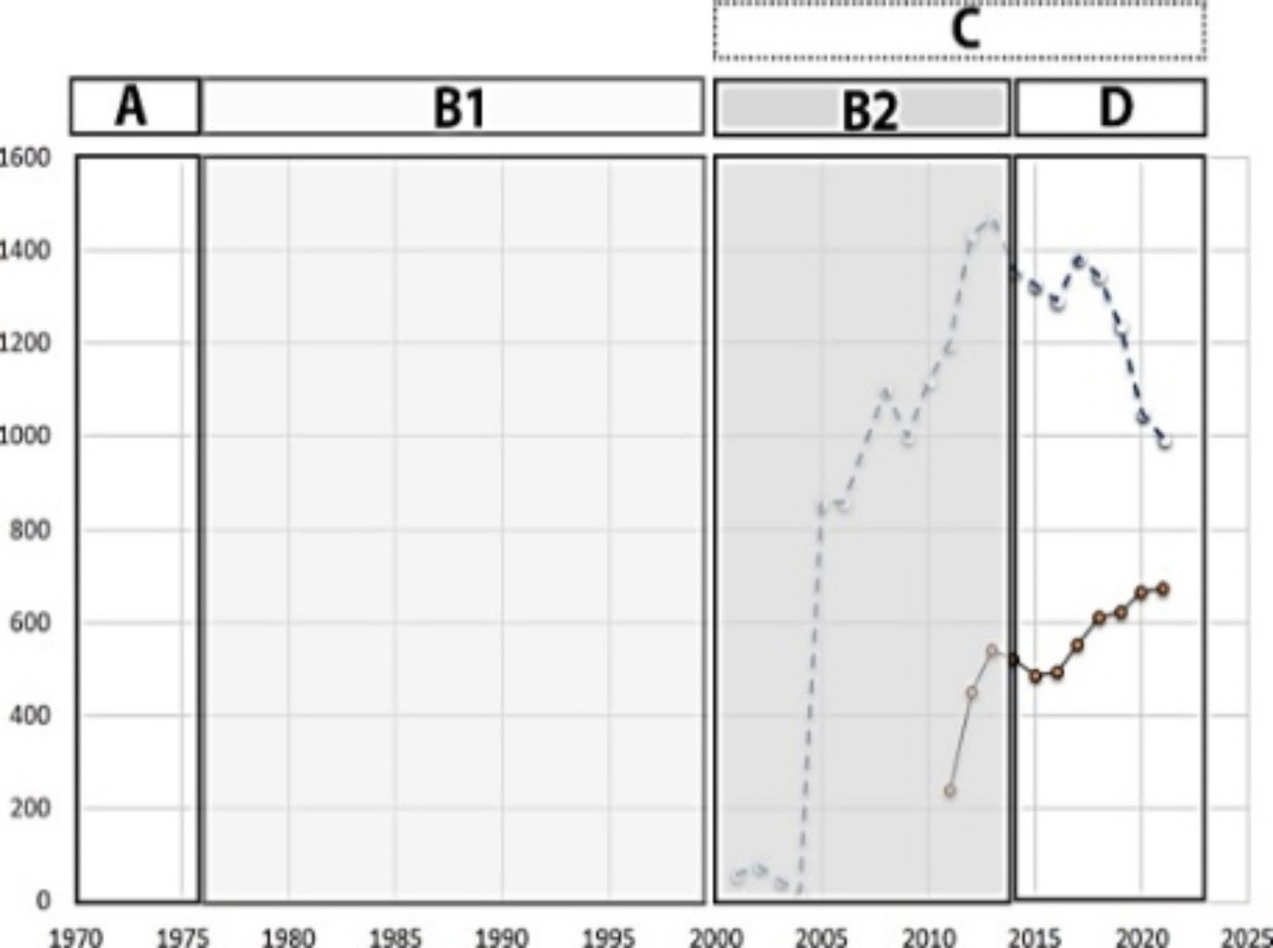
Frequency distribution of EMS in the last century

To better understand the situation, the frequency distribution plot of EMS according to the last-twenty-year trend was exploited (Fig. 2). Here, the horizontal axis represents time (in year) and the vertical axis shows frequency (viz., number of people). As well, the dotted curve and the continuous line in black display the frequency of Associate’s and Bachelor’s degrees in Pre-Hospital EMS, respectively. Additionally, four time situations are marked with letters from A to D (with black and white columns). Each letter also stands for a situational map of the concepts associated with the role of EMS. To obtain such concepts at each time period, the messy situational maps are initially drawn, and then the ordered ones, plotted by Clarke (2018), are used (Table 3). In these maps, the human, non-human, discourse, and other components in this situation along with their connections are described.

**Table 3:** SA Ordered Situational Map for EMS In Iran

Individual Human Elements/Actors	Nonhuman Elements/Actants
Nurses LPN Patients Faculty Hospital managers Nursing managers Faculty in nursing & EMS Health system policymakers	Development of nursing knowledge Advances in Medical Sciences Medical equipment Medications and treatment methods Increase library resources Development of medical specialization Specialized expectations in bed
**Collective Human Elements/Actors**	**Major Issues/Debates (Usually Contested)in EMS**
Ministry of Health (Vezarat-e Behdari)in the past (1952–1986), Ministry of Health and Medical Education Since 1986, Iranian Nursing Association (1921–1978), Nursing Board (since 1991), Health Department and Treatment of the National Military System (IRGC) The Deputy of Nursing Iranian Nursing Organization (INO) (2003) Iranian Medical Emergencies Association (2000) Medical Emergencies Board (1999 till now) Emergency Information Centers (1975) Pre-hospital emergency centers and incident management 1357 until now) Emergency Organization of the country (since 2015) Red Crescent Relief and Rescue Organization of the country Red 1929 until now Fire Department (1921 until now)	Shortage Of EMS Higher Education In EMS Establishment Iranian Nursing Organization (INO) for standardization practice The wandering of the field of emergency medicine in the organization chart of the faculties (uncertainty of the field in the nursing or paramedical faculty) Mechanisms of presence of women in the field of emergency medicine Lack of leveling of care among emergency medical forces Not having a medical emergency representative in the country's nursing system organization Continuous and non-continuous undergraduate studies in emergency medicine Air medical services
**Economic Elements**	**Sociocultural/Symbolic Elements**
Sales of Iranian oil and improvement of financial situation in the years (1340 to 1351) Private sector investment in the health system	Village and urban population Cultural revolution in the country's higher education system Women's participation in society woman enter the EMS profession
**Economic Elements in health care system**	**Political Elements**
the creation of educational infrastructure in the field of nursing and LPN employ faculty members in nursing the quantitative growth of nursing Recruitment of academic staff in nursing (1983 so far), health management in disasters and medical emergencies and emergency medicine Increase absorption in emergency medical admissions Setting up private ambulances (2013 so far)	Iran's membership in the World Health Organization The royal family's support for the role of EMS in society Islamic Revolution(1) The Iraq - Iran War (1978–1989) Establishment of Islamic Azad University
**Temporal Elements**	**Related Discourses (Historical, Narrative, and/or Visual)**
The Iraq - Iran War (1978–1989)	The Diminished Involvement Of Women And Volunteers In Nursing (1961–1980**)** The Active Presence Of Affluent And Sometimes Middle-Class Women In The Social The Engagement Of Women, Especially From The Low And Middle-Class Class In Social Activity
**Implicated/Silent Actors**	
need of population for health services	

### Situational map

#### A. EMS emergence in Iran (1970–1975)

Before 1973, there was no formal education program for EMS in Iran. According to the available documents, the collapse of the roof of the main hall of the MIA terminal building, where 16 people lost their lives and 11 individuals were injured, was the stimulus to establish EMS in Iran. After that, a team of Iranian physicians were sent to the United States to study EMS, who then returned after six months with two highly skilled EMS educators, Jim Patterson and Max. Following a public call for people having a high school diploma and holding an entrance exam, they selected the best and employed them as EMS technicians, completing an intensive six-month training course. Therefore, EMS officially started in 1975 under the title, Emergency Information Center (EIC) [25]. As highlighted in one of the interviews, Participant No. 2, a pre-hospital EMS pensioner, said that:

…at that point in time, you could simply take part in such courses with all types of high school diplomas. My hometown had three centers for the whole province, and there were three ambulances. As I remember, another support was a rescue worker who was also an ambulance driver…

#### B. EMS quantitative growth and infrastructure in Iran

Based on the interviews and the existing documents, the EMS equipment and infrastructure, particularly the physical settings, developed at three time periods as follows:

##### **B1. Establishing EMS centers and supplying personnel (1976–2002)**

Starting new political relations with other countries, including those in the Point Four Program (as the United States policy announced by President Harry S. Truman in 1949) and the Central Treaty Organization (CENTO), between 1948 and 1978, as well as membership in the WHO, overshadowed Iran’s situation with the urgency of change. In this global situation, corresponding to the Second, Third, and Fourth Economic Development Plans (EDPs), Iranian oil exports instigated economic growth. Such political and economic changes in the government accordingly pave the ground for the establishment of EMS infrastructure [26, 27], so that it started in 1975 in this country, which was ranked fourth after the United States, Canada, and Australia [28]. Based on the study findings, Iran was among the most advanced nations in terms of medical equipment and the fleet of ambulances at that time. During the Islamic Revolution (1978) and in the course of the Iran-Iraq War (1980–1987), the EMS personnel educated in the previous years had to serve in such crises.

The EMS resources and equipment have been thus exploited in Iran for about thirty years (1975–2002). As well, higher public awareness and the prevalence of NCDs (such as, heart attacks, strokes, diabetes, etc.) gradually raised the need for specialized personnel for primary care and patient transfer, which demanded a platform for the scientific development of pre-hospital EMS.

At this period, due to the severe shortage of personnel across Iran, the healthcare system focused on supplying EMS personnel to provide the required services. Currently, after about half a century, more than 20000 EMS personnel and experts, with Associate’s and Bachelor’s degrees, are working in INMEO, but this is far from the standard indicators in proportion to the growing population, the fleet of ambulances, and EMS centers [29].

In this sense, Participant No. 9 stated that:

*“…. There were only three emergency center and five American ambulances in Mashhad, which were used less due to high fuel consumption… “*

##### **B2. Improving medical equipment, renewing the fleet of ambulances, and smartening EMS (2002–2016)**

With the onset of making EMS a subject of academic study in Iran (2002), medical equipment was improved and the fleet of ambulances was renewed. In 1999, the first Air medical emergency center launched in Tehran. As well, the Communication and Operation Command Center (COCC) (that is, the Dispatch Center) was organized in a more specialized manner [30]. During the last two decades, the renovation of the fleet of ambulances became a priority in INMEO. At present, the standard indicator is one ambulance for every 20000 people. INMEO also has 3000 land centers, including 1700 road centers and 1300 urban centers, 50 Air medical emergency centers, two railway lines, and 300 motorlances [15].

**Fig. 3:**
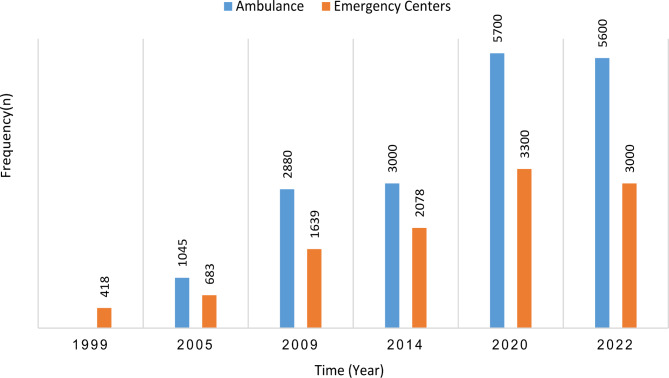
The number of ambulances and emergency centers in Iran in the last twenty years

From the start of the EMS in Iran, receiving the missions was in a traditional (wireless), analog form. Following technological advancement and the digitalization of Iran’s healthcare system, the algorithm of telephone triage, ambulance management, and patient information documentation changed due to the use of a software program, called ASAYAR, but the previous system was maintained for further support [31] (Fig. 3).

In this sense, Participant No. 6 stated that:

*… “We had heard that there were light helicopters in the emergency that were used in very rare cases ….”*

#### C. EMS clinical education development in Iran (2002–2023)

**Fig. 4:**
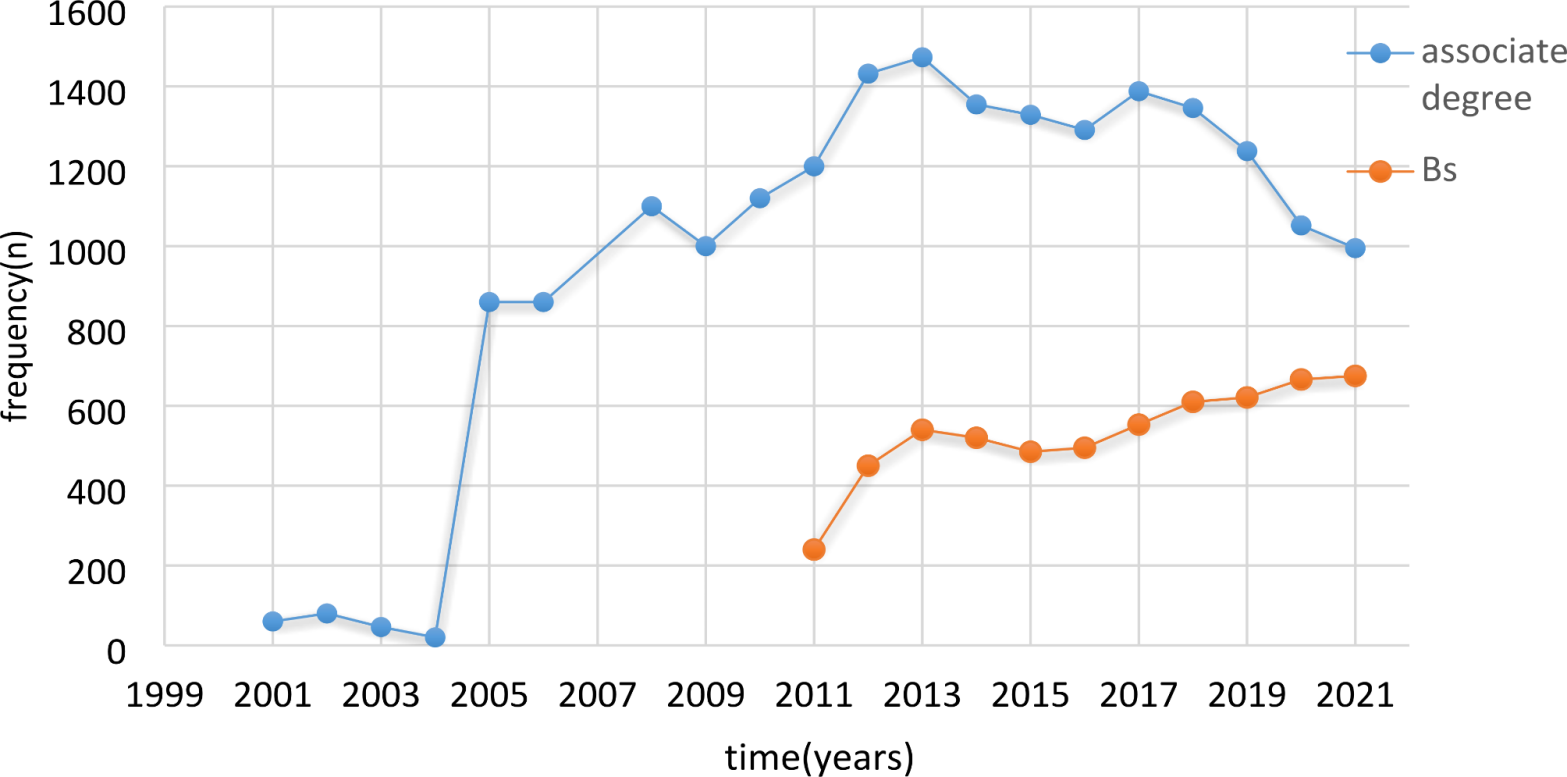
Distribution of the frequency of admission to the national entrance exam for pre-hospital emergency medicine in the last two decades of Iran

With the merger of the universities of medical sciences and their affiliated hospital in Iran, the former Ministry of Health (MOH) was formed under the name, Ministry of Health, Treatment, and Medical Education (MOHTME) in 1986 [12]. Accordingly, nursing education changed following the transformation of nursing institutes into schools and colleges. For about three decades (1975–2002), the EMS education was in the form of a six-month course for volunteers with all types of high school diplomas at the schools of nursing. In addition, the rescue workers who had completed the courses held by the Iranian Red Crescent Society (IRCS) were also working as drivers in INMEO. The curriculum for the Associate’s program in EMS was thus compiled in 1999, and approved in the eighth meeting of the Supreme Council for Medical Sciences Planning (SCMSP) at MOHTME [31]. Since 2002, the EMS education system entered into a new stage by accepting students for Associate’s degree programs in EMS in Iran. University education was further improved in 2011 by accepting 240 candidates applying for a non-continuous Bachelor’s degree. At present, male and female students are being accepted for Associate’s and non-continuous/continuous Bachelor’s degrees [32]. The point to consider in the EMS clinical education development is the lack of a platform for postgraduate studies. In this regard, standards and job descriptions of different nursing groups were compiled with the official establishment of INO (2003). In addition, the start of INMEO, specialty boards, and IEMSA led to the legal differentiation of the role of EMS from relief and rescue. During the quantitative growth (Fig. 4) and the development of EMS clinical education, the rescue workers were further removed from the pre-hospital EMS. For the first time in Iran, in 2020, female students were accepted in the field of emergency medicine in several universities of medical sciences. Currently, most of the female graduates are working in the communication and operations management center and in the operational centers for women in the centers of the provinces.

In this sense, Participant No. 6 stated that:

*“… From 1995 onwards, the type of activities became more and more advanced and there was a lot of focus on learning intubation in the system, and in the way that there are currently protocols and instructions and sometimes the right to prescribe medicine, it was not like that at that time. The type of activities moved towards becoming more professional, and in general, our dealings with clients have become more professional and ethical …”*

#### D. Specialization of role of pre-hospital EMS (2014-Present)

Upon the graduation of the pre-hospital EMS personnel, holding academic degrees, the healthcare and clinical services became gradually more scientific and specialized. The activities of the EMS personnel accordingly moved from simple and basic tasks, such as primary care and patient transfer to more advanced scientific and specialized ones, i.e., primitive and advanced CPR, working with equipment such as an electroshock and an automated external defibrillator (AED), telemedicine, coding patients with heart attacks and strokes, etc. [27]. This led to a qualitative growth in the role of EMS. In this sense, Participant No. 4 stated that:

…we were in charge of transferring the patients at that time. We thus went to Jorjani School for taking a short six-month course, and then entered the emergency department due to a severe shortage of personnel. These days, those who are coming here have university education and academic degrees. Some equipment such as AED is available in our base, and we have learned to work with them…

### Social worlds/arenas maps

In these maps, the actions of people both as individuals and as members of social worlds could be examined in the current discourses. To plot it, the collective entities or social worlds in the arenas could be thus addressed, so that the collective agents and arenas of commitment and discourse could be further analyzed in a situation [33]. The historical trend of collections and organizations related to pre-hospital EMS in Iran indicated them as social worlds in local, national, and international organizations that might have an effect on the role of EMS, such as MOH in the past (1952–1986), MOHTME since 1986, the EMS policymakers in EIC (from 1975 to 1991), IEMSA (2000 to present), MEAMC (1991 to 2016), INMEO (2016 to present), the EMS specialty boards (1999 to present), the nursing boards (from 1999 to present), and the health and treatment departments of the military system (i.e., Guards Corps and the Army), IRCS (1929 until now), Fire Department (1921 until now), and IRCS Relief and Rescue Organization.

The influential people of these organizations in the government system accordingly have bureaucratic and supervisory power to develop programs and perform roles in the healthcare field. Currently, MOHTME is the heart of funding EMS education, which is more important than that for other organizations.

**Fig. 5:**
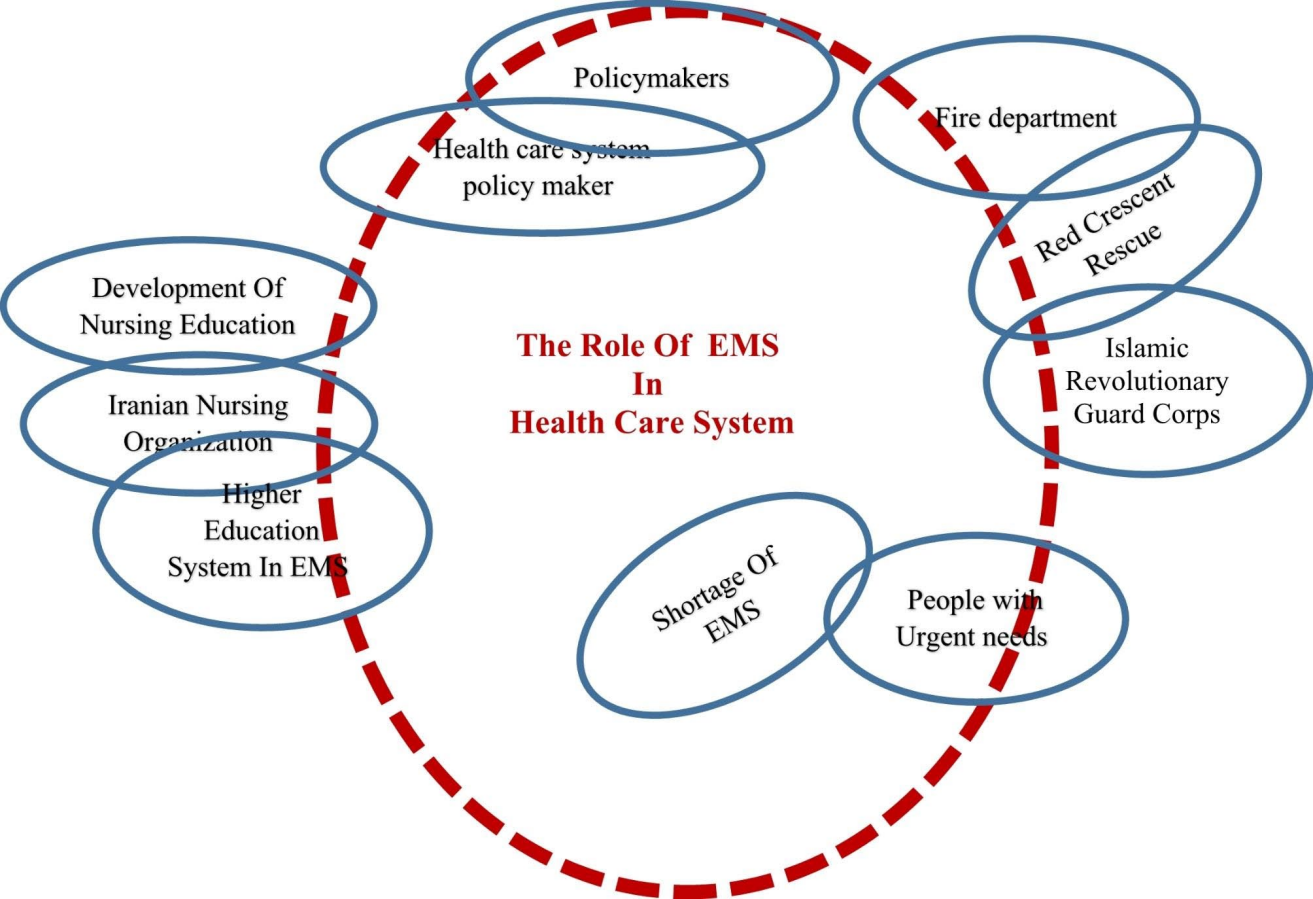
clarke’ social worlds/ arenas Map: The trend of changes in the role of Emergency Medical Services

Another key issue in the related organizational groups is the close and direct relationship between the Fire Department, IRCS R&RO, and Iran’s National Police Force (INPF) and the pre-hospital EMS personnel. In such a way, the Fire Department puts out the fire, releases the injured inside the city, and IRCS R&RO helps in natural disasters to rescue the injured on the road [34]. This between-organization interaction can have a significant impact on EMS time indicators and performance in providing services to clients (Fig. 5).

### Positional maps

Positional maps illustrate a deep understanding of the data in the adopted and unadopted positions, which are organized based on the differences, conflicts, and concerns about various issues [33]. The key positions affecting the trend of change in the role of EMS are thus as follows:

A. Prevalence of NCDs, indicating the high demand for EMS in the society
B. Specialization of pre-hospital EMS
C. Lack of equipment and resources (namely, personnel, funding, etc.)

There is also a shortage of EMS personnel with academic degrees in Iran, so that the national standard is one urban base with one active ambulance for every 20000 people and one road base with one operating ambulance in each city with a population of less than 20000. Besides, the number of standard personnel in each base is 9 people. The official statistics released by INMEO have also filed 1300 urban centers for 63 million urban population and 1700 road centers. This shows the lack of 3150 urban centers and 3300 road centers for the current position of Iran to be close to the national standards [35]. Therefore, Iran’s healthcare system lacks human resources and equipment for EMS provision, coming from several reasons, viz., no recruitment, disproportionate geographic distribution and dispersion of EMS personnel across the country, outdated organizational charts, etc.

The above positional map displays that the healthcare system policymakers working on service provision, particularly in the pre-hospital field, need to make operational decisions to improve the quality of care to clients by categorizing different levels of pre-hospital care and changing the standards, to be compatible with the complexities of current expected care and the new economy of systems.

## Discussion

It is clear that the role of EMS in Iran and the world has undergone significant changes over the years due to various factors, including political relations, establishment of laws and regulations, crises, education development, technological advancement, and infrastructure improvements [36, 37].

The emergence of EMS in Iran was initially due to the collapse of the roof of the main hall of MIA terminal building in 1973. However, for about thirty years, there were no significant changes in EMS education or medical equipment, and no academic education was established for pre-hospital EMS. After the Islamic Revolution and the Iran-Iraq War, there was a small growth in the number of rescue workers, and the dire need for more advanced nursing and healthcare services during the war paved the ground for pre-hospital EMS [38].

However, it was not until after 2002 that Iran began to create a more suitable situation for changes in the role of EMS by developing pre-hospital EMS education and renovating the fleet of ambulances and medical equipment [3, 39].

One significant factor affecting the role of EMS was the development of EMS education, which led to the graduation of pre-hospital EMS personnel with academic degrees, making clinical care more scientific [22, 40–42]. Another factor was the smartening of pre-hospital infrastructure, with the use of digital technology to improve the algorithm of telephone triage, ambulance management, and patient information documentation. This led to an improvement in Iran's time indicators in providing EMS to clients [43].

It is worth noting that the structure and composition of Iran's pre-hospital EMS are different from that of other countries, with two people present in the role of EMS personnel and a physician providing aids via phone calls and consultations, while in other countries, pre-hospital EMS is met by a team of physicians, EMS personnel, and nurses. This distinction can have a significant effect on the expected EMS type and performance [17, 30, 44, 45, 46, 47].

Like in many other countries, pre-hospital EMS personnel in Iran face a range of challenges. Some of these challenges include: Lack of resources: Despite recent improvements in the fleet of ambulances and medical equipment, there is still a lack of resources in some areas, especially in rural and remote regions [3, 36, 48]. Limited access to continuing education: While there have been improvements in pre-hospital EMS education in Iran, access to continuing education and training opportunities can still be limited in some areas [36, 49, 50, 51]. Inadequate compensation: Pre-hospital EMS personnel in Iran may not be adequately compensated for their work, which can lead to low morale and high turnover rates [3, 52, 53]. Safety concerns: Pre-hospital EMS personnel in Iran may face safety concerns due to the nature of their work, including exposure to infectious diseases, physical violence, and traffic accidents [48, 50, 52]. Language barriers: In some areas of Iran, pre-hospital EMS personnel may face language barriers when communicating with patients and their families, which can make it difficult to provide appropriate care [49, 50, 53]. Cultural and social norms: Cultural and social norms in Iran can also pose challenges for pre-hospital EMS personnel, particularly when it comes to providing care to women and addressing sensitive medical issues [40, 48].

## Conclusion

Like other countries in the world, the role of EMS in Iran became vital due to the prevalence of NCDs, trauma triage in accidents, crises, and the high demands in the society; however, the roots of inadequate EMS and the related reasons in Iran have their own similarities and differences with those in the world.

Considering the trend of change in the approaches adopted by healthcare systems across the world, and given the breakthroughs in nursing and medicine, along with the education of professionals during the last thirty years, the descriptions of duties and performance in EMS has moved from primary care and patient transfer to outpatient care and specialized services. However, sufficient EMS infrastructure has not been developed in terms of postgraduate education. In addition, the cultural context specific to Iran, the challenges of women working in EMS centers, the disconnection of service providers, namely, IRCS R&RO, INPF, and INMEO, lack of resources and equipment, and the geographical distribution of human resources based on population dispersion, are thus among the significant issues facing pre-hospital EMS in this country.

### Suggestions and implications

To make more progress and reach a balanced development in the role of EMS, there is a need for a scientific review of the high-level courses of pre-hospital EMS, in parallel with the clarity of the role and the position of the Associate’s and Bachelor’s degrees as an urgency of change, which should be established by analyzing all situational maps. Given the economic pressure and the lack of EMS, continuously affecting Iran’s healthcare system, nursing managers and policymakers should attempt to strengthen the legal structures, executive regulations, and the standards appropriate to Iran’s situation as soon as possible to develop the role of EMS. Furthermore, there is a need to keep thinking about pre-hospital EMS teams in new situations.

### Limitations

Getting permissions to interview the top-level managers in Iran’s healthcare system was not possible in this study, so they were not recruited. Additionally, some archives and library documents could not be reviewed due to their historical age.

## Data Availability

The datasets generated in the current study are available from the corresponding author upon reasonable request.

## List of abbreviations

EMS: Emergency Medical Services
SA: Situational analysis
NCDs: Non-Communicable Diseases
IRCS: Iranian Red Crescent Society
INMEO: Iran’s National Medical Emergency Organization
INPF: Iran’s National Police Force
HRs: Human resources
CPR: Cardiopulmonary resuscitation
WHO: World Health Organization
GT: Grounded theory
MEAMC: Medical Emergency and Accident Management Center
IEMSA: Iranian EMS Association
NLAI: National Library and Archives of Iran
INO: Iranian Nursing Organization
EIC: Emergency Information Center
CENTO: Central Treaty Organization
EDPs: Economic Development Plans
COCC: Communication and Operation Command Center
AED: Automated External Defibrillator
INPF: Iran’s National Police Force
